# Susceptibility weighted imaging depicts retinal hemorrhages in abusive head trauma

**DOI:** 10.1007/s00234-013-1180-7

**Published:** 2013-04-09

**Authors:** Giulio Zuccoli, Ashok Panigrahy, Anshul Haldipur, Dennis Willaman, Janet Squires, Jennifer Wolford, Christin Sylvester, Ellen Mitchell, Lee Ann Lope, Ken K. Nischal, Rachel P. Berger

**Affiliations:** 1 Department of Pediatric Radiology, Children’s Hospital of Pittsburgh of UPMC, 4401 Penn Avenue, Pittsburgh, PA 15224 USA; 2 Division of Child Advocacy, Children’s Hospital of Pittsburgh of UPMC, 4401 Penn Avenue, Pittsburgh, PA 15524 USA; 3 Eye Center, Children’s Hospital of Pittsburgh of UPMC, 4401 Penn Avenue, Pittsburgh, PA 15224 USA; 4 Division of Pediatric Ophthalmology, Strabismus, and Adult Motility, Eye Center, Children’s Hospital of Pittsburgh of UPMC, Pittsburgh, PA USA; 5 Safar Center for Resuscitation Research, University of Pittsburgh Medical Center, Pittsburgh, PA USA; 6 Department of Radiology, Children’s Hospital of Pittsburgh of UPMC, 4401 Penn Avenue, Pittsburgh, PA 15209 USA

**Keywords:** Child abuse, Eye hemorrhage, Retinal diseases, Magnetic resonance imaging, Domestic violence

## Abstract

**Introduction:**

This study aims to evaluate the capability of magnetic resonance imaging (MRI) susceptibility weighted images (SWI) in depicting retinal hemorrhages (RH) in abusive head trauma (AHT) compared to the gold standard dilated fundus exam (DFE).

**Methods:**

This is a retrospective, single institution, observational study on 28 patients with suspected AHT, who had a DFE and also underwent brain MRI-SWI as part of routine diagnostic protocol. Main outcome measures involved evaluation of patients to determine whether the RH could be identified on standard and high-resolution SWI sequences.

**Results:**

Of the 21 subjects with RH on DFE, 13 (62 %) were identified by using a standard SWI sequence performed as part of brain MRI protocols. Of the 15 patients who also underwent an orbits SWI protocol, 12 (80 %) were positive for RH. None of the seven patients without RH on of DFE had RH on either standard or high-resolution SWI. Compared with DFE, the MRI standard protocol showed a sensitivity of 75 % which increased to 83 % for the orbits SWI protocol.

**Conclusions:**

Our study suggests the usefulness of a tailored high-resolution orbits protocol to detect RH in AHT.

## Introduction

Multiple population-based studies report the rate of abusive head trauma (AHT) to be markedly high. The incidence of severe or fatal AHT in infants less than 1 year old was 29.7 per 100,000 according to a population-based study in North Carolina [1]. Several other studies had documented even higher rates in Pennsylvania and New York [2, 3]. While retinal hemorrhages (RH) can also occur in children with non-abusive traumatic brain injury (TBI), they occur in less than 10 % of cases of non-abusive TBI and are generally limited to the posterior pole and to a single layer of the retina. In contrast, children with AHT often have much more extensive RH which extend out to the ora serrata and occur in multiple layers of the retina [4]. Hence, identification of the RH, and their extent, can be important in the diagnosis of AHT. It is often not possible to safely dilate the eyes in a child with an acute TBI because of the concern that while the eyes are dilated, the treating team will lose the ability to evaluate pupillary dilatation, an important clinical tool for the assessment of neurologic status. Accordingly, the eye exam may be often delayed for several hours or even a day or two, or inadequate, if performed through the undilated pupil. This can be problematic for several reasons. First, superficial or preretinal RH can resolve very quickly, sometimes within 24 h [4]. There is also some evidence to suggest that in very ill children, precisely the ones in whom it may be unsafe to dilate the eyes acutely, there may be an increase in the amount of hemorrhage or even an occurrence of new hemorrhage early in the hospitalization [5]. Finally, in the first few days after a severe TBI, children may develop medical complications such as a coagulopathy or increased intracranial pressure. While these medical conditions do not cause the type of RH seen in children with AHT, whether or not they could cause a worsening of the RH is unknown, although the issue is often raised in the legal setting [4]. Issues of safety are also confounded by logistical impasses, as a pediatric ophthalmologist is not always available in the 24 h after a child is admitted for suspected AHT. An alternative method to identify RH could therefore be very helpful.

The susceptibility weighted images (SWI) sequence is a gradient-echo imaging technique to amplify the signal of the tissues with magnetic susceptibility differences relative to the background, including partially deoxygenated venous blood, clot, calcium, and iron-laden tissue [6–8]. SWI is broadly used in trauma, tumors, multiple sclerosis, stroke, hemorrhagic conditions, vascular malformations, and metabolic disorders [9]. Small and large vascular structures as well as micro bleeds may be depicted by SWI images. SWI may be three to six times more sensitive than conventional T2*-weighted gradient-echo sequences in depicting the size, number, volume, and distribution of hemorrhagic lesions in diffuse axonal injury [10]. Although gradient-echo images demonstrated extensive RH in one case report [11], the SWI sequence has not been previously been used for the evaluation of RH.

Hence, magnetic resonance imaging (MRI) could play a role in early detection of RH in cases where the gold standard dilated fundus exam (DFE) cannot be performed for aforementioned reasons. The goal of this preliminary study was to evaluate the usefulness of standard and high-resolution orbits SWI protocol in the detection of RH in children with suspected AHT.

## Methods

Our study group was a consecutive sample of 28 patients with suspected AHT who underwent both a brain MRI and DFE by a pediatric ophthalmologist at Children Hospital of Pittsburgh of UPMC as part of clinical care from March 1, 2011 to June 1, 2012.

### Ethics statement

The study was approved by our Institutional Review Board with a waiver of informed consent as part of a larger study related to the identification and evaluation of AHT.

### Ophthalmological evaluation

Written documentation of the DFE was used to collect the following data: side of RH (right, left, or both), extent of RH (posterior pole or beyond the posterior pole), and layers of the retina (preretinal, intraretinal, and subretinal). A quantitative evaluation of the RH was obtained from the DFE analysis. In each case, photos of the RH were taken using a RetCam (Clarity MSI, Pleasanton CA). Patients with less than or equal to five RH on DFE were considered to have “few RH.” Patients with more than five RH were considered to have “several RH.”

Timing of the DFE was taken as the number of days between the MRI and the eye exam was also collected: positive numbers referred to subjects in whom DFE was performed before the MRI and negative numbers, when the MRI was performed first.

### MRI

MRI was conducted on a 1.5T magnet in 22/28 (78 %) of subjects and on a 3T magnet (Signa, HD platform HDxt 16.0, GE Healthcare, Milwaukie, WI) in 4/28 (14.2 %) patients by using a standard brain protocol that included a brain SWI sequence. In 15 (53.5 %) of the patients, a high-resolution orbits SWI protocol was also obtained to examine the orbits. For the standard SWI sequence, the image parameters were as follows: repetition time/echo time (TR/TE) = 50.0/78.3 ms, slice thickness 3 mm, field of view (FOV) = 200 mm, flip angle = 15, matrix 288 × 224, in-plane resolution = 0.6 mm on the 1.5T magnet; and TR/TE = 46.9/26.0 ms, slice thickness 3 mm, FOV = 200 mm, flip angle = 15, matrix = 320 × 224, in-plane resolution = 0.6 mm on the 3T magnet, respectively. For the SWI high-resolution orbits protocol, the image parameters were as follows: TR/TE = 50.0/78.3 ms, slice thickness ranging from 1 and 1.4 mm, FOV = 200 mm, flip angle = 15, matrix = 288 × 224, in-plane resolution = 0.5 mm on the 1.5T magnet; and TR/TE = 46.6/26.0 ms, slice thickness = 1 mm, FOV = 200 mm, flip angle = 15, matrix = 320 × 256, in-plane resolution = 0.6 mm on the 3T magnet, respectively. For the standard MRI brain protocol, the patients underwent the SWI sequence using images with a 3-mm slice thickness that specifically included the orbits. The patients studied with the high-resolution orbits SWI protocol were also evaluated using images with a 1-mm slice thickness. The SWI images were qualitatively analyzed for the presence or the absence of RH by two neuroradiologists blinded to the result of the DFE (GZ and AP). RH were defined as areas of low signal intensity along the retina demonstrated on SWI on more than one single slice. Imaging analysis was confined to the axial plane.

## Results

Twenty-eight children were enrolled with a mean age of 10.9 months, with a standard deviation of 7.2. The majority of the patients were males 19/28 (67.9 %). Patient race was not available.

### Timing of MRI and DFE

For 5 subjects, the DFE was done prior to the MRI, 5 had both exams done the same day, and 18 subjects had the MR imaging done after to the DFE.

### Ophthalmological findings on DFE

RH were identified by DFE in 21/28 (75 %) patients. Demographics, DFE, and MRI findings from these 21 patients are shown in Table 1. Of the patients with RH, 13/21 (61.9 %) had RH localized to the posterior pole; 8/21 (38.1 %) had RH which extended beyond the posterior pole.

**Table 1:** Demographics, dilated fundus exam, and magnetic resonance imaging findings

	Sex/age	Right eye DFE		Brain SWI	Orbit SWI	Left eye DFE		Brain SWI	Orbit SWI
		Number	Location			Number	Location		
1	M/11	>5	I	Pos	Pos	>5	P–I	Pos	Pos
2	M/6	>5	I	Neg	NA	>5	I	Neg	NA
3	M/5	>5	P–I	Pos	NA	>5	P–I	Pos	NA
4	M/2	>5	P–I	Pos	NA	>5	P–I	Pos	NA
5	F/5	>5	P–I–S	Pos	NA	>5	P–I–S	Neg	NA
6	M/3	>5	P–I	Neg	Pos	>5	P–I	Pos	Pos
7	M/9	≤5	I	Neg	Pos	≤5	P–I	Neg	Pos
8	M/2	>5	P	Pos	Pos	>5	P–I	Pos	Pos
9	M/5	>5	P–I	Pos	Pos	>5	P–I	Pos	Pos
10	M/16	>5	I	Neg	Pos	≤5	I–S	Neg	Pos
11	M/22	>5	P–I–S	Pos	Pos	>5	P–I–S	Pos	Pos
12	M/23	≤5	I	Neg	Neg	≤5	I	Neg	Neg
13	M/14	≤5	I	Neg	NA	≤5	I	Neg	NA
14	M/24	>5	P–I–S	Neg	NA	>5	P–I–S	Neg	NA
15	M/5	≤5	I	Pos	Pos	NA	NA	Neg	Neg
16	F/7	>5	P–I–S	Pos	Pos	>5	P–I–S	Neg	Pos
17	F/25	≤5	I–S	Neg	Neg	≤5	I–S	Neg	Neg
18	F/8	>5	P–I–S	Pos	Pos	≤5	P–I–S	Pos	Pos
19	F/22	≤5	I	Neg	Neg	NA	NA	Neg	Neg
20	F/17	≤5	P–I–S	Pos	Pos	≤5	P–I–S	Neg	Neg
21	F/4	≤5	I	Pos	Pos	≤5	I	Neg	Pos

Age (in months); >5 and ≤5 indicate the number of retinal hemorrhages

*M* male, *F* female, *DFE* dilated fundus exam, *I* intraretinal hemorrhages, *P* preretinal hemorrhages, *S* subretinal hemorrhages, *Brain SWI* standard brain MRI-SWI protocol, *Orbit SWI* dedicated SWI high-resolution protocol

### MRI findings

**Fig. 1:**
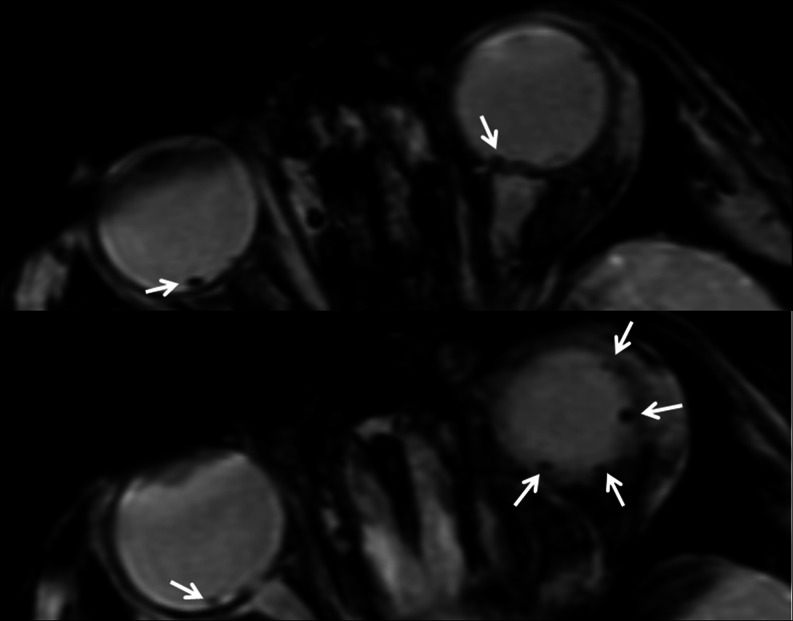
MRI on patient 1. Multiple areas of low signal intensity consistent with retinal hemorrhages are noted on SWI images (*arrows*) on a 3T magnet on the standard brain MRI protocol. Intraretinal hemorrhages were identified in the right fundus, and intraretinal and preretinal hemorrhages were identified in the left fundus on DFE (not shown)

**Fig. 2:**
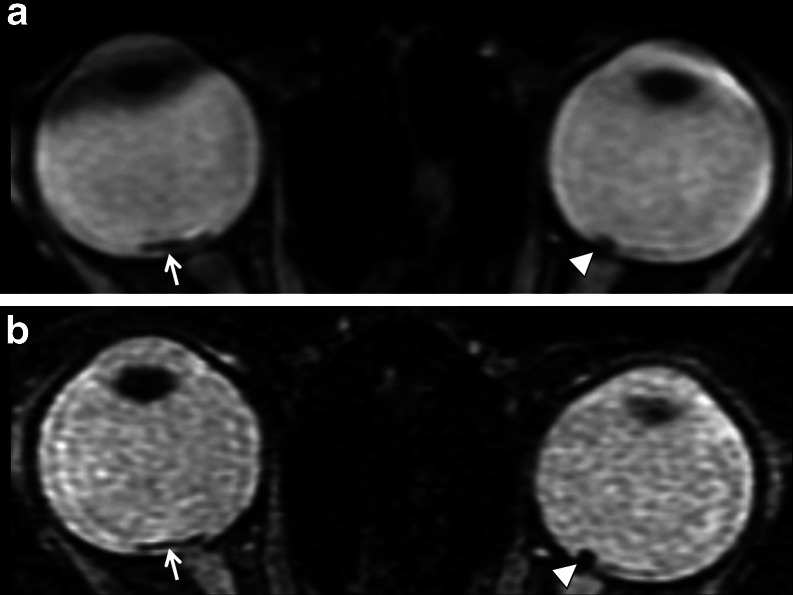
MRI on patient 6. Areas of low signal intensity consistent with hemorrhages are noted on SWI images on a 1.5T magnet on the standard brain MRI-SWI protocol (**a**) and on the dedicated high-resolution orbits SWI protocol (**b**). The hemorrhages are better delineated on high-resolution images (**b**). Other RH was also identified (not shown)

**Fig. 3:**
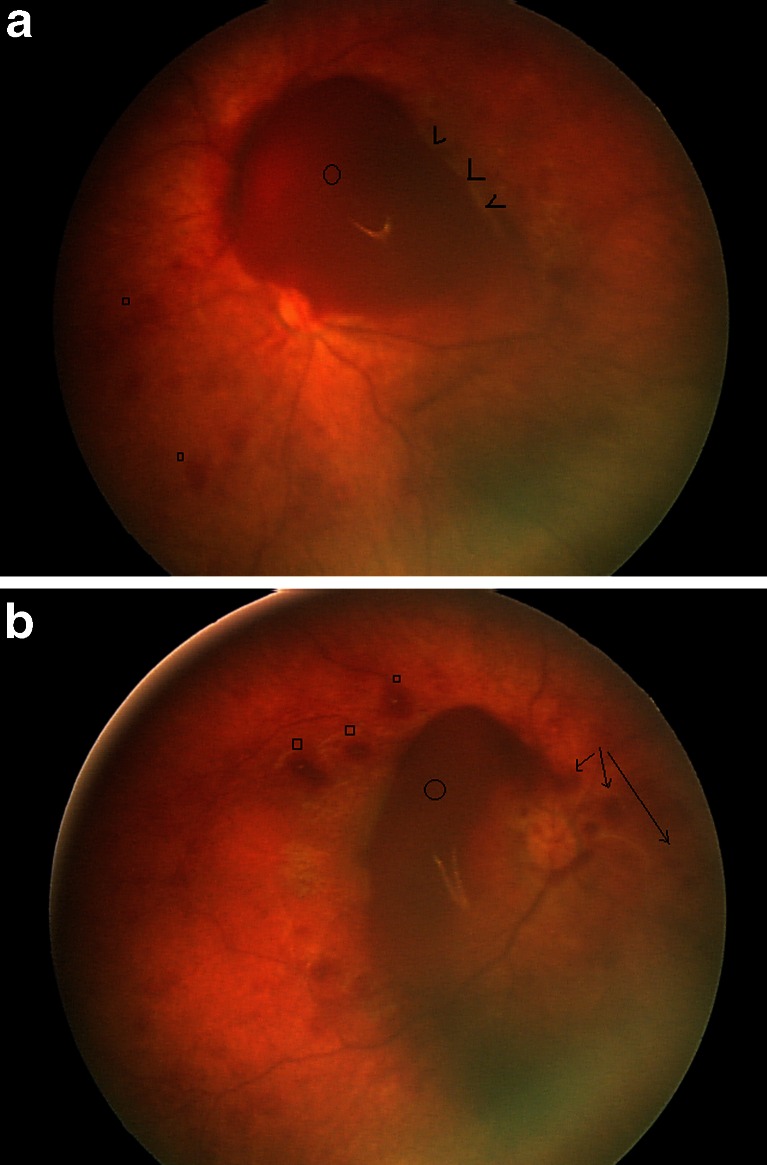
RetCam pictures of patient 6. **a** Right fundus. There are white-centered superficial intraretinal hemorrhages (*squares*) and deep intraretinal hemorrhages (*arrowheads*). There is a large preretinal hemorrhage obscuring part of the optic disk (*circle*). **b** Left fundus. There are some superficial intraretinal hemorrhages (*squares*) and less obvious deep intraretinal hemorrhages. There is a larger preretinal hemorrhage obscuring the optic disk (*circle*). The suggestion of some schitic retinal changes is felt to represent an artifact of the imaging system (*arrows*)

Of the 21 subjects with RH on DFE, 13 (62 %) were identified using SWI in the setting of the standard brain MRI protocol. Of the 15 patients with RH on DFE who also underwent a dedicated high-resolution orbits SWI protocol sequence, RH was identified in 12 (80 %). In the three patients in whom high-resolution SWI did not depict RH, there were less than or equal to five RH on DFE in each case (patients 12, 17, and 19; Table 1). Therefore, the standard SWI protocol showed a sensitivity of 75 %, while the high-resolution orbits SWI protocol had a sensitivity of 83 %. The specificity was 100 % for both SWI protocols. Figures 1 and 2 show the MRI findings in patients 1 and 6. Figure 3 shows the RetCam pictures from patient 6.

## Discussion

This is the first study utilizing a 3D high-spatial-resolution SWI protocol to depict RH. In our patient population, the high-resolution orbits SWI protocol was able to identify RH in 80 % of the subjects compared to the 62 % of the subjects evaluated with the standard brain MRI protocol, with a higher sensitivity demonstrated by the high-resolution orbits SWI protocol over the standard brain MRI protocol—75 over 83 %, respectively—suggesting that a high-resolution orbits SWI protocol may be uniquely adept at detecting such hemorrhages. In those patients with a positive DFE and a negative high-resolution orbits SWI protocol, only few retinal hemorrhages were identified on DFE, supporting the notion that the detectability threshold for RH could be lowered by a further increase in the resolving power of the SWI sequence. Imaging of retinal hemorrhages in cases of suspected abusive head trauma is an essential part of documentation. Until now, fundus cameras that have been linked to digital indirect ophthalmoscopes, or have a pan retinal capture with contact of the camera with the cornea, have been used. Non-photographic methods have not been reported very much in the literature. CT scan and ultrasound do not appear to have been systematically used or studied for detection rates of the types of hemorrhages seen in AHT. Recently, however, findings from handheld spectral domain optical coherence tomography in shaken baby syndrome have been reported. These authors concentrated in detecting on the vitreo–retinal interface rather than on all the hemorrhages present [12]. As far as we are aware, the use of SWI to assess retinal hemorrhage has not previously been reported.

### Potentials and limitations of SWI images in the evaluation of the retina

SWI is a volumetric three-dimensional sequence tailored to be particularly susceptible to signal heterogeneities within the magnetic field, induced by paramagnetic or diamagnetic materials, hemosiderin, and calcium among the others. The signal intensity from paramagnetic materials grows (“blooms”) on SWI sequences. When compared with conventional gradient-echo (T2*) sequences, SWI will enhance small changes in susceptibility across a voxel as signal intensity losses. On SWI images, the higher the resolution, the lower the blooming artifact. This is likely to improve the morphological delineation of paramagnetic and diamagnetic materials. In contrast, non-hemorrhagic paramagnetic or diamagnetic materials as dystrophic calcifications could be mistakenly identified as RH on SWI.

## Conclusions

A DFE by a pediatric ophthalmologist is the gold standard evaluation for the assessment of RH. However, DFE is not always available or safe in the acute care setting. Our study suggests that there may be an advantage to integrate a dedicated high-resolution orbits SWI protocol in the neuroimaging work-up of pediatric patients being evaluated for AHT. Prospective studies are warranted to support our preliminary data.

## References

[1] Keenan HT, Runyan DK, Marshall SW (2003). A population-based study of inflicted traumatic brain injury in young children. JAMA.

[2] Dias MS, Smith K, DeGuehery K (2005). Preventing abusive head trauma among infants and young children: a hospital-based, parent education program. Pediatrics.

[3] Berger RP, Fromkin JB, Stutz H (2011). Abusive head trauma during a time of increased unemployment: a multicenter analysis. Pediatrics.

[4] Levin AV (2010). Retinal hemorrhage in abusive head trauma. Pediatrics.

[5] Gilles E, McGregor M, Levy-Clarke G (2003). Retinal hemorrhage asymmetry in inflicted head injury: a clue to pathogenesis?. J Pediatr.

[6] Reichenbach JR, Venkatesan R, Schillinger DJ (1997). Small vessels in the human brain: MR venography with deoxyhemoglobin as an intrinsic contrast agent. Radiology.

[7] Haacke EM, Xu Y, Cheng YC (2004). Susceptibility weighted imaging (SWI). Magn Reson Med.

[8] Tong KA, Ashwal S, Obenaus A (2008). Susceptibility-weighted MR imaging: a review of clinical applications in children. AJNR Am J Neuroradiol.

[9] Tong KA, Ashwal S, Holshouser BA (2003). Hemorrhagic shearing lesions in children and adolescents with posttraumatic diffuse axonal injury: improved detection and initial results. Radiology.

[10] Tong KA, Ashwal S, Holshouser BA (2004). Diffuse axonal injury in children: clinical correlation with hemorrhagic lesions. Ann Neurol.

[11] Altinok D, Saleem S, Zhang Z (2009). MR imaging findings of retinal hemorrhage in a case of nonaccidental trauma. Pediatr Radiol.

[12] Muni RH, Kohly RP, Sohn EH, Lee TC (2010). Hand-held spectral domain optical coherence tomography finding in shaken-baby syndrome. Retina.

